# Treatment of Fresh Fractures of the Leg by Massage and Passive Movements

**Published:** 1903-09

**Authors:** David E. Wheeler

**Affiliations:** Buffalo, N. Y.; 564 Delaware Ave


### Treatment of Fresh Fractures of the Leg by Massage and Passive Movements.

BY DAVID E. WHEELER, M. D., Buffalo, N. Y.

TEXTBOOKS on surgery inform us that from six to eight weeks are required for the repair of a fracture of the leg, but our patients tell a different story. By the time bony union is complete the troubles of the surgeon may be over, but those of the patient are just beginning. When the limb is taken from plaster, it is stiff, thin and weak, while edema of the skin shows its poor nutrition. These sequelæ are especially marked in periarticular fractures. Our treatment has cured the bony lesion,—now the patient must cure the results of treatment; voluntary or passive motion must restore the function lost by immobilisation.

1. Read before the Erie County Medical Association, March 9, 1903.

A distinction must here be drawn between the weakness and atrophy directly caused by prolonged constriction and immobility, and that sometimes caused by the original traumatism, due to the laceration of muscles and the rupture of ligaments and tendons. But even this second kind of weakness is better treated by means directed to maintaining the nutrition of the limb, than by prolonged and absolute rest. Still more important is the distinction between permanent stiffness, caused by vicious union with exuberant callus, and the temporary stiffness caused by immobilisation during and after the repair of traumatic synovitis and the other common complications of simple fractures. Obviously our eagerness to prevent the temporary evil should not lead us to risk the permanent one.

So the problem confronts us,—can we prevent malnutrition of the soft parts without endangering the correct repair of the bony lesion? This question was answered in the negative, and the disagreeable sequelæ of immobilisation considered inevitable in fracture of the leg until Lucas-Championniére proved it possible to get good results without retentive apparatus. He discarded splints entirely, except for special indications, and treated most of his fractures from their inception by daily rubbing the parts and moving the joints about them. These movements were on no account to be voluntary by the patient, but passive and applied by the surgeon. He called attention to the facts that ribs and collar-bones healed rapidly with good functional results, that function was more important than form and that in delayed union it had always been considered good treatment to stimulate repair by rubbing the bone ends together. He reported good results from his treatment. The contraindications he gave were: (1) extreme tendency to malposition; (2) large blebs; (3) overriding; (4) fragments in such a position or of such shape as to easily work through the skin and compound the fracture.

Championniére claimed not only to be able to avoid all the sequelæ of fracture, but also to shorten the time required for bony union by one-third,—the same treatment which favored nutrition in the soft parts, improved the circulation in the bone and so hastened the process of repair. His results have been confirmed by the observers using his methods, although they have not been as extreme as he in discarding all retentive apparatus. Following Championniére's publication a number of surgeons, mostly German or French, reported good results from his method of treatment somewhat modified. The German surgeons gave their fractures one massage treatment immediately following reduction and then immobilised them for from four to fourteen days in plaster-of-paris, using as a guide to the duration of immobilisation, the length of time needed to give sufficient union to prevent recurrence of deformity when manual support was substituted for the splint. As soon as this could be safely done the leg was massaged daily for from fifteen to twenty minutes, and at the same time passive motion cautiously applied. Between treatments the leg was kept in a Volkman or other similar splint. The results obtained in this way by the German surgeons were: patients able to walk with crutch or cane in, on the average, fourteen days; total treatment, average duration twenty-four days.

The French surgeons followed Championniére pretty closely, but for the most part used splints between the applications of massage. Caldwell and Wiener, of Chicago, and Woolsey, of New York, have reported good results from the massage treatment of fractures.

During a part of the time that Dr. Woolsey, as visiting surgeon on the first surgical division of Bellevue Hospital, was collecting the material for his papers, I was interne on the second surgical and, therefore, had a good opportunity to employ his method and to see as well as read of his results. Besides other cases he reports 47 of Pott's fracture, in which injury he finds massage especially indicated. His treatment is as follows: the fracture is reduced and put up in a Volkman splint or plaster-of-paris if that is necessary to maintain reduction. As soon as massage and passive movements can be borne without pain, if the deformity does not recur when the leg is gently lifted from the splint, he gives daily seances of massage, lasting for about a quarter of an hour. At the end of each active treatment he returns the leg to the splint. As long as the patient remains in bed the splint is used between sessions. In the 47 cases of Pott's fracture reported, the patients were confined to bed from 14 to 33 days, the average being 18 days. Generally, when first out of bed, these patients walked with a crutch and later with a cane, although exceptionally they could from the first walk freely without artificial aid. The average duration of treatment was 25 days, and at their discharge from the hospital all could walk without support and only five limped.

Dr. Woolsey always used sufficient apparatus to prevent malposition. If a solid plaster-of-paris splint was found necessary for the first 10 days, he found the results nearly as good as in those cases where massage was used from the first; but, if, on the contrary, massage was used for the first one to three weeks and it was then found necessary to place the leg in plaster for two weeks, the result was very little better than if plaster was used during the whole treatment. He advises, therefore, in case of doubt, complete immobilisation at the start.

While Dr. Woolsey was using this treatment on the first surgical division, I obtained permission from Dr. Hotchkiss, visiting surgeon on the second surgical, to try it in our wards. The cases treated comprised all the commoner fractures of the leg and the exact method used was as follows: if there was very little swelling or acute pain, as in fractures of the fibula alone,—massage and passive movements of the joints were instituted at once and repeated daily. The leg was immobilised between the massage treatments as long as there could be elicited a false point of motion. On the other hand, if there was considerable swelling, or the pain was acute, or large blebs were present, massage was deferred until these symptoms had subsided; rest, meanwhile, being secured by means of a Volkman splint. For excessive swelling an ice cap was used with or without a wet lead and opium dressing. The blebs were pricked and dusted with boric acid powder. Usually in from one to four days massage could be borne without much pain. The leg was then removed daily from the splint and treated for about 20 minutes by deep gentle pressure, with the tips of the fingers and the palm of the hand immediately above and below the point of fracture, but not directly on the solution of continuity. These massage movements were entirely from the extremity and towards the heart. At the same time the ankle- and knee-joints were flexed and extended. Great care was exercised that these manipulations should not be made with sufficient force to cause palpable movement at the point of fracture. Often at first it was impossible to remove the leg from the Volkman splint without causing deformity. In these cases the bandage was cut down daily and the front of the leg massaged, but it was not raised from its support, nor were flexion and extension practised until the formation of provisional callus was sufficiently firm to prevent movement of the fractured bone ends on one another during these manipulations.

At Bellevue, both on the first and second divisions, massage was performed by the nurses, but unless a nurse can be procured who is exceptionally familiar with fractures this should be done by the surgeon, or a competent assistant. Certainly no one should be trusted to massage fresh fractures unless competent to detect deformity, reduce it and apply the apparatus necessary to keep it reduced.

It was found impossible to maintain good position in some of the fractures with a Volkman splint, or any other splint disturbed daily. Therefore, these cases were kept in a permanent solid plaster-of-paris dressing until consolidation was far enough advanced to permit of massage without eliciting a false point of motion. These were mostly fractures of both bones of the leg, in which injury early massage can only be used for those near the ankle and occasionally for transverse fractures of the upper and middle thirds.

Generally, in from one to two weeks from the time of fracture the patients were allowed to go about on crutches. When this was done the leg was protected not by a Volkman, but by a posterior plaster-of-paris splint; in Pott's fracture supplemented by a strip running from the instep over the outer border of the foot, under the sole and up the inner side of the leg,—Stimson's dressing. At first all cases were allowed and urged to walk with neither crutch nor cane as soon as they could do so without pain,—that is in about two weeks,—and were discharged from the hospital a few days after they could walk. They were, however, asked to return once a month for inspection. The later cases on the contrary, except fractures of the fibula alone, were not allowed to rest the weight of the body on the broken leg until the end of the fourth, fifth or even sixth week.

The cases treated were as follows: fractures of both bones of the leg, 22; Pott's fracture, 20; fracture of the fibula alone, 13; fracture of the tibia alone, 2. Total, 57.

I shall now review the results obtained in—(*a*) Fracture of the fibula alone; (*b*) Pott's fracture; (*c*) Fracture of the tibia, with or without fracture of the fibula.

(*a*) Fractures of the fibula alone were usually able to walk without pain in from 9 to 14 days. They were then allowed to do so and did not require either crutch or cane. They showed neither limp, pain, nor weakness,—function was completely restored and union firm in two weeks or even less. They were discharged from the hospital as soon as they wished. Many of them returned to report their condition after a month or more, and not one of those returning showed any untoward result from this early use of the leg.

(*b*) Pott's fractures were able to walk at the end of the second or third week and, although they limped somewhat when first using the leg, function was completely restored by a few days use. The early cases were urged to walk unaided as soon as they could, but of those allowed full use of the leg under four weeks some who returned for inspection suffered from recurrence of deformity and partial loss of function. From this the conclusion was reached that even under massage treatment, if a patient is allowed to walk on a Pott's fracture within two weeks of its receipt, he may get a deformed and crippled ankle. This weakening of the arch of the foot did not occur until the leg had been in use several weeks, and so might be easily overlooked in hospital patients. On this account the later cases were not allowed to bear weight on the fracture, even although anxious to do so until the end of the third, fourth or fifth week. This delay is likely to be very irksome, since it is very hard for a man to hobble about on crutches several weeks after the cessation of all subjective symptoms, but it should, I think, be insisted upon, or the final result is endangered. Of the cases not allowed to walk until after the twenty-eighth day quite a few returned for inspection and not one showed recurrence of deformity, weakening of the arch of the foot, or any loss of function. The delay did not impair the functional result at the time of discharge from the hospital. Four or five weeks after the receipt of injury Pott's fractures walked home with no stiffness and very little weakness.

(*c*) *Fractures of the tibia.*—In these cases early massage was used only in fractures near the ankle or in those where the line of cleavage was squarely transverse,—that is, in those showing comparatively little tendency to overriding or other deformity. When of such a nature that they could be treated early by massage, they showed at the end of two weeks complete absence of pain, even on voluntary movements of the knee or ankle, or when the heel was struck a sharp blow. They refused to walk, however, saying the "leg felt too weak", and on close examination a false point of motion could generally be elicited at the site of fracture. They were gotten out of bed, however, and went about on crutches; meanwhile, the leg was protected by a posterior plaster splint and massage continued. They were able and willing to walk by the end of the fourth week, but after seeing some cases of recurring deformity in Pott's fractures allowed to walk too early they were not allowed to do so until the end of the fifth or sixth week. After union is too firm to allow any false motion and walking is painless, at least one week should elapse before the patients are allowed full use of the limb.

None of this third class of cases walked before the end of the fourth week and not one who returned for inspection showed deformity sufficient to be noticeable, or to cause any impairment of function.

### CONCLUSIONS.

1. Fractures of the fibula alone may be fearlessly treated by massage and passive movements from their inception, and those so treated may be allowed to walk two weeks after the receipt of fracture without compromising the final result.

2. In Pott's fracture and fractures involving the tibia more caution should be used. The patients will often require a solid plaster-of-paris splint for the first 10 days, which will, of course, preclude the use of massage during that time. They should not be allowed to walk for at least a week after bony union is apparently firm. This will make the total time of disability from 28 to 42 days. At no time during treatment should any deformity be tolerated and our power to prevent deformity is a distinct limit to the applicability of this method of treatment.

3. The time required for true bony union cannot be much shortened.

4. The total time of disability is, however, materially shortened and the final result as to function is as good, if not better, than under strict complete and prolonged immobilisation. This gain is obtained rather by preventing sequelæ than by hastening union.

5. The traditional ideas concerning the amount and duration of immobilisation necessary to secure good union in fractures of the leg need revision.

Immobilisation is a distinct detriment to the patient, and no more of it should be used than is found in each case to be required to give correct apposition of the broken bone ends. As soon as examination shows that apparatus can be discarded without causing recurrence of deformity, this should be done without waiting to comply with any rules for the duration of immobilisation. In most cases the slight disturbance caused by frequent inspections of a fracture is beneficial rather than injurious.

### REFERENCES.

Donvrain: Sur le Traitement des Fractures par le Massage et la Mobilisation. Merville. 1898.

Lucas-Championnière: Traitement des Fractures par le Massage et la Mobilisation. Paris. 1895.

Petit: Quelques cas de Fracture Traitées par le Massage. Bordeaux. 1887.

Angelesco: Arch. de Soc. Médicale de Bucharest. Paris. 1897. II., p. 207.

Bennett: Lancet. London. 1898. I., p. 359.

A. Bum: Wiener Klinik. 1895. XXI., p. 20.

Buscarlet: Rev. Med. de la Suisse Rom. Genève. 1897. P. 752.

Caldwell: Railway Surgeon. Chicago. 1898-99.. V. 515.

Davis: Ann. Surg. Philadelphia. 1896. XXIV., 691.

Krecke: München Med. Wochenschrift. 1892. P. 214.

Hamel: Bl. f. Klin. Hydrotherap. Wien. 1898. P. 98.

Lucas-Championnière: Bull et Mem. de chir. de Paris. 1886. XII., p. 560. Med. News. New York. 1900. LXXVI., p. 1-3.

Labbè: Bulletin Acad. de Med. Paris. 1897. XXXVIII., p. 695.

Miller: Practitioner. London. 1897. LVIII. P. 264.

Reclus: Gaz. Hebd. de Med. Paris. 1890. P. 26.

T. A. Selenkow: St. Petersberg Med. Wochenschrift. 1889. No. 6.

Wanscher: Gaz. d Hôp. Paris. 1893. LXVI. P. 959.

Wiener: Chicago Med. Recorder. 1896. P. 96.

Woolsey: Ann. Surgery. Sept., 1900. XXXII; Medical News. Vol. LXX. March 20, 1897.

564 Delaware Ave.

